# GERO Cohort Protocol, Chile, 2017–2022: Community-based Cohort of Functional Decline in Subjective Cognitive Complaint elderly

**DOI:** 10.1186/s12877-020-01866-4

**Published:** 2020-11-25

**Authors:** Andrea Slachevsky, Pedro Zitko, David Martínez-Pernía, Gonzalo Forno, Felipe A. Court, Patricia Lillo, Roque Villagra, Claudia Duran-Aniotz, Teresa Parrao, Rodrigo Assar, Paulina Orellana, Carolina Toledo, Rodrigo Rivera, Agustín Ibañez, Mario A. Parra, Christian González-Billault, Helena Amieva, Daniela Thumala

**Affiliations:** 1 Geroscience Center for Brain Health and Metabolism (GERO), Santiago, Chile; 2 Neuropsychology and Clinical Neuroscience Laboratory (LANNEC), Physiopathology Department - Institute of Biomedical Sciences (ICBM), Neuroscience and East Neuroscience Departments, Faculty of Medicine, University of Chile, Santiago, Chile; 3 Memory and Neuropsychiatric Clinic (CMYN) Neurology Department, Hospital del Salvador and Faculty of Medicine, University of Chile, Santiago, Chile; 4 Department of Neurology and Psychiatry, Clínica Alemana-Universidad del Desarrollo, Santiago, Chile; 5 Department of Neurosciences, Faculty of Medicine, Universidad de Chile, Santiago, Chile; 6 Health Service & Population Research Department, IoPPN, King’s College London, London, UK; 7 Escuela de Salud Pública, Universidad de Chile, Santiago, Chile; 8 Center for Social and Cognitive Neuroscience (CSCN), School of Psychology, Universidad Adolfo Ibáñez, Santiago, Chile; 9 Center for Integrative Biology, Faculty of Sciences, Universidad Mayor, Santiago, Chile; 10 The Buck Institute for Research on Aging, Novato, USA; 11 South Neurology Department, Faculty of Medicine, University of Chile, Santiago, Chile; 12 Unidad de Neurología, Hospital San José, Santiago, Chile; 13 East Neurology Department, Faculty of Medicine, University of Chile, Santiago, Chile; 14 Facultad de Psicología, Universidad Alberto Hurtado, Santiago, Chile; 15 Institute of Biomedical Sciences (ICBM), Faculty of Medicine, University of Chile, Santiago, Chile; 16 Neuroradiologic Department, Instituto de Neurocirugia Asenjo, SSMO, Santiago, Chile; 17 Cognitive Neuroscience Center (CNC), Universidad de San Andrés, Buenos Aires, Argentina; 18 National Scientific and Technical Research Council (CONICET), Buenos Aires, Argentina; 19 Universidad Autónoma del Caribe, Barranquilla, Colombia; 20 Global Brain Health Institute (GBHI), University of California San Francisco (UCSF), California, USA; 21 Psychology Department, School of Psychological Sciences & Health, University of Strathclyde, Glasgow, UK; 22 Department of Biology, Faculty of Sciences, Universidad de Chile, Santiago, Chile; 23 INSERM, Bordeaux Population Health Research Center, UMR 1219, Univ. Bordeaux, F-33000 Bordeaux, France; 24 Escuela de Psicologia, Facultad de Ciencias Sociales, University of Chile, Santiago, Chile

**Keywords:** Cognitive aging, Subjective cognitive complaint, Dementia, Alzheimer, Functional decline, Geroscience

## Abstract

**Background:**

With the global population aging and life expectancy increasing, dementia has turned a priority in the health care system. In Chile, dementia is one of the most important causes of disability in the elderly and the most rapidly growing cause of death in the last 20 years. Cognitive complaint is considered a predictor for cognitive and functional decline, incident mild cognitive impairment, and incident dementia. The GERO cohort is the Chilean core clinical project of the Geroscience Center for Brain Health and Metabolism (GERO). The objective of the GERO cohort is to analyze the rate of functional decline and progression to clinical dementia and their associated risk factors in a community-dwelling elderly with subjective cognitive complaint, through a population-based study. We also aim to undertake clinical research on brain ageing and dementia disorders, to create data and biobanks with the appropriate infrastructure to conduct other studies and facilitate to the national and international scientific community access to the data and samples for research.

**Methods:**

The GERO cohort aims the recruitment of 300 elderly subjects (> 70 years) from Santiago (Chile), following them up for at least 3 years. Eligible people are adults not diagnosed with dementia with subjective cognitive complaint, which are reported either by the participant, a proxy or both. Participants are identified through a household census. The protocol for evaluation is based on a multidimensional approach including socio-demographic, biomedical, psychosocial, neuropsychological, neuropsychiatric and motor assessments. Neuroimaging, blood and stool samples are also obtained. This multidimensional evaluation is carried out in a baseline and 2 follow-ups assessments, at 18 and 36 months. In addition, in months 6, 12, 24, and 30, a telephone interview is performed in order to keep contact with the participants and to assess general well-being.

**Discussion:**

Our work will allow us to determine multidimensional risks factors associated with functional decline and conversion to dementia in elderly with subjective cognitive complain. The aim of our GERO group is to establish the capacity to foster cutting edge and multidisciplinary research on aging in Chile including basic and clinical research.

**Trial registration:**

NCT04265482 in ClinicalTrials.gov. Registration Date: February 11, 2020. Retrospectively Registered.

**Supplementary Information:**

The online version contains supplementary material available at 10.1186/s12877-020-01866-4.

## Background

Population ageing, driven by rising life expectancies and declining fertility rates, is one of the most important transformations the world is undergoing today. World population over 60 years old is now 12% and is expected to reach 21.5% by the year 2050. Within the same period, the increase in the population over 80 years old will be even more pronounced, going from 1.7 to 4.5% of the population [1]. This demographic change is advancing faster in Latin America (LA) than in European and North American countries [2, 3]: by 2025, the total number of individuals over 60 years old will reach approximately 57 million [4]. Among the countries in this region, Chile shows one of the fastest life expectancy growth rates [2, 3, 5]. By 2050, Chileans older than 60 years will increase from the current 15.7% of the population to 32.9%, while people older than 80 years will reach 10.3% [5].

This population ageing is associated with a strong increase in the number of people living with dementia, which is estimated to reach 140 million by 2050. Dementia is the most significant global challenge for health and social care in the twenty-first century [6]. In Chile, dementia is the leading cause of dependency (36%) in older people [7–9]. The National Survey of Dependency in the Elderly reported an estimated prevalence of dementia of 7.0% (women 7.7%, men 5.9%) in people aged 60 years and older [7]. This prevalence is equivalent to what is reported in a systematic review of epidemiologic studies of dementia in Latin America [10]. In addition, the number of deaths attributed to dementia in Chile has increased by 526% from 1990 to 2010, which means that dementia is the most rapidly growing cause of death in the last 20 years [11].

Most of the dementia syndromes are preceded by a prodromal phase characterized by the presence of a broad range of very subtle manifestations of cognitive decline. Common presentations are, amongst others, concerns about cognitive decline, also known as subjective cognitive complaint (SCC), of people who may or may not have deficits in objective testing [12], reported either by the person her/his-self, or by an informant, mild cognitive impairment (MCI), and the recently proposed mild behavioral impairment [13]. Although many subjects with SCC and MCI are at high risk to progress to a dementia syndrome (i.e. conversion rates to dementia range from 2 to 15% per year in subjects with MCI), some of them remain stable over time while others revert to healthy cognition, particularly in epidemiological settings [14–16]. This uncertain prognosis makes cognitive complaints and MCI important construct in terms of targeting interventions for secondary prevention in dementia [14, 17].

On the other hand, studies focused on the risk factors associated with functional decline (FD), i.e. the ability to perform daily routines, are less known. The determination of FD has been commonly used as a critical line dividing between predementia and dementia stages. However, the notion that FD starts only at the stage of dementia has been challenged with several studies showing that minor impairment in complex activities of daily life (ADL) precedes dementia in many years [18, 19], and is already present at the stage of MCI [20, 21]. Moreover, standardizing the degree of functional impairment that is associated with dementia rather than MCI has been problematic and the categorical classification of MCI and Alzheimer’s Disease (AD) has been criticized [22]. Studying the amount and trajectories of FD could allow overcoming limitations associated with categorical outcomes, such as the conversion to dementia. Moreover, impairment in the ability to perform everyday activities and the eventual loss of independence are major concerns for older adults [23].

Finally, predicting the risk of FD and the risk of dementia is associated with a complex interplay of non-modifiable and modifiable risk factors such as overall health and lifestyle factors [24]. The interplay of these factors could be divided into five dimensions: i) biologic, ii) neuroimaging, iii) clinical phenotype (cognition, behavioral, motor and functional domains), iii) metabolic, systemic diseases and habits and v) psychosocial. A multidimensional assessment including all dimensions mentioned above would increase either the differentiation of healthy and pathologic brain aging and the prediction of the risk of FD and dementia [25].

To the best of our knowledge, no previous study has reported multidimensional risk factors (biomedical, imaging, psychosocial, and clinical) associated with the prognosis of elderly with SCC on the evolution of FD. Also, there is a scarcity of cohort study on cognitive decline in Latin-American and no studies have been carried out in Chilean on risks associated with progression to dementia. The present paper reports the aims and design of a cohort study, being conducted in Chile by the Geroscience Center for Brain Health and Metabolism (GERO).

### Objectives of the study

The general objective of this study is to analyze the rate of functional decline and progression to clinical dementia and their associated risk factors (biomedical, imaging, psychosocial, and clinical) in a community-dwelling elderly with SCC, through a population-based study. The specific objectives are to determine: i) longitudinal evolution of biomarkers measured from blood, stool and structural and functional magnetic resonance neuroimaging (MRI), ii) evolution of health-related outcomes, including quality of life, comorbidity and risk factors, and iii) mortality rates. We also aim to build the capacity to undertake clinical research on brain ageing and dementia disorders and to create data and biobanks with the appropriate infrastructure to conduct other studies and facilitate to the national and international scientific community access to the data and samples for research.

The GERO cohort is the core clinical project of the GERO program grant, which is supported by the Fund for Research Centers in Priority Areas Program (FONDAP) of the Chilean national research and development agency (ANID, for its acronym in Spanish). GERO is initially funded for 5 years, and its main aim is to establish a center for studying brain aging in Chile, including basic and clinical research.

## Methods/design

### Setting

The cohort recruits the potential participants from the general population, using a door-to-door strategy. The sample framework corresponds to the territories assigned to three primary healthcare centers selected by convenience according to their socioeconomic heterogeneity, which belong to three different districts in Santiago (Chile): Macul, Peñalolen and La Reina. The sample considers a two-stage selection process. The first stage includes a sample of quadrants within each territory, where the contact to all houses is attempted. The second stage proceeded when in a home is found more than one potential eligible participant, choosing one randomly. Territories encompassed a population between 14,937 and 39,458 people [26], of which between 4.6 and 8.0% is expected to be older than 70 years old. Follow up of the participants is performed in the Memory and Neuropsychiatry Clinic (CMYN, for its acronym in Spanish) at the Universidad de Chile, located next to the Hospital Salvador, hospital of reference for territories included in the sample.

### Participant, eligibility, inclusion, and exclusion

Subjects are eligible for the study if they fulfil the following criteria: i) 70 years old or older; ii) presence of a knowledgeable informant and/or presence of a contact that allows the follow up of the participant, and iii) being affiliated to the public health insurance.

Eligible participants are invited to the study and receive a first evaluation to confirm the following criteria:

Inclusion criteria: Eligibility criteria plus:

- Subjective cognitive complaint either self-reported or reported by a knowledgeable informant.
- Clinical Dementia Scale— frontotemporal lobar degeneration (CDR-FTLD) equal or inferior to 0.5 [27].
- Signed informed consent.

Exclusion criteria:

- Report of medical diagnosis of dementia.
- Mini-mental State Examination (MMSE) < 21 and Pfeffer questionnaire > 2 [28, 29].
- Institutionalization (for example, living in an elderly home or a skilled nursing facility).
- Illiteracy, meaning that is not able to write or read.
- Visual and auditory acuity not adequate for neuropsychological testing.
- Important limitation of mobility incompatible with the availability to be independent in daily life activities and/or attending a clinical center for further evaluation.
- Report of medical diagnosis of Parkinson’s disease.
- Report of medical diagnosis of one or more of the following conditions causing severe impairment in functionality: any psychiatric or neurological disorders, brain tumor, subdural hematoma, progressive supranuclear palsy, or history of head trauma.
- Report of medical diagnosis of stroke occurred in the last 3 months.
- Presence of a fatal disease (less than 1 year of survival).

### Field work during the first contact

**Fig. 1:**
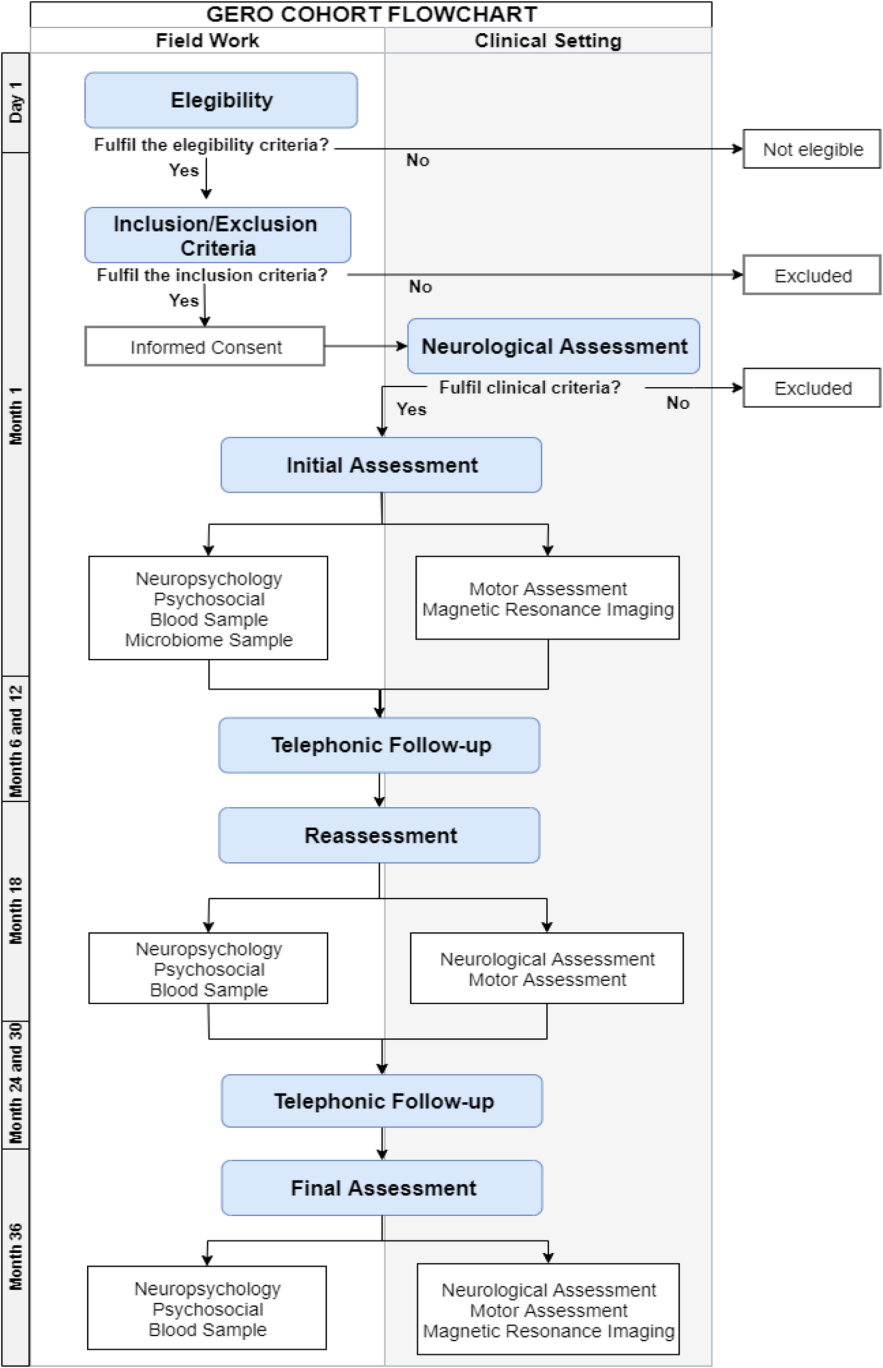
Flow Chart of Study Procedure

The recruitment process considers two steps. First, a lay team contacts each home to determine the presence of eligible individuals. In positive cases, the person receives a second visit by a trained psychologist who proceeds to check for eligibility. In case of acceptance, the inclusion and exclusion criteria protocol are applied. If the subject fulfils the criteria, the psychologist schedules a medical interview. Following this evaluation, a neurologist decides if the subject fulfil the inclusion criteria of the cohort (see Fig. 1).

The fieldwork is preceded by an outreach campaign (flyers, local radio advertisements, and presentations to community-organized groups) raising awareness about the visit of interviewers and the relevance of participating in the study. Rates of contact and response are monitored permanently, and the procedures around the contact and first interview are checked in the field and also by telephone to a subsample of the participants. Contact to homes is attempted up to three times on different days and hours before considering it frustrated. The fieldwork started in November 2017 and is expected to finish at the middle of 2020. Up to date, the recruitment has not been completed.

The lay team and psychologists involved in the first contact and recruitment received specific training on their labor in the field. The lay team completed a whole training week, which included theoretical and practical elements. Psychologists received a 12 weeks length training, which covers several sessions of neuropsychological assessment.

### Sample size

The sample size needs to satisfy two criteria, one concerned with the statistical power required to explore multiple associations with outcomes, and other related to the feasibility to perform a wide range of assessments to each participant assuming costs and logistics. Both criteria meant a trade-off between the tolerance to uncertainty around the parameters to be estimated and the number of assessments that would be investigated throughout the study. The final sample chosen was 300 participants. This number allows maintaining the integrity of the original protocol and permits to test associations equivalent to an Odds Ratio (OR) around 1.5 (Cohen’s equal to 0,22) in cases of exposition and probability of the outcome close to 50%, using a significance of 5%. It is expected to follow each participant 3 years, accumulating roughly 900 person-years of follow up.

### Follow-up and retention strategy

Socio-demographic, health-related outcomes (quality of life, arthrometric measures and risk factors), clinical stages and symptoms, psychosocial, neuropsychological, neuropsychiatric, motor, neuroimaging, blood biomarkers, stool, and genetic samples will be performed as baseline evaluation and every 18 months, with the exception of the genetic study that will be performed only at baseline and neuroimaging at baseline and 36 months. Patients’ health status, functionality, and involvement in the GERO cohort will be monitored every 6 months by a telephonic questionnaire in order to assess general well-being and keep contact with the participants.

To avoid a significant attrition of the sample the following strategies have been considered: to recruit only people who have at least one person that can facilitate the contact with him or her, it means a person who can be contacted for asking about the location of the participant; telephone contact every 6 months; and domicile visit in case of absence of contact or attending to assessment appointments. Additionally, all transport costs of participants are being covered by the GERO cohort administration, as well as any food that is required during the days of assessment. Initially, the end of the follow up of the cohort is programmed for October 2022.

### Assessments and measurements

The protocol considers an intensive and deep multidimensional study of factors related to the prognosis of FD and dementia development. The range of assessments includes: socio-demographic, health-related outcomes (quality of life, arthrometric measures and risk factors), clinical stages and symptoms, psychosocial, neuropsychological, neuropsychiatric, motor, neuroimaging, blood biomarkers, genetic and stool samples to perform gut microbiome studies (see Table 1, Fig. 1 and Additional file 1). Neuroimaging protocol will allow assessing brain atrophy, structural and functional connectivity and white matter lesions [83, 84] (see Additional file 1). GERO biological samples of whole blood, buffy coat, plasma, serum, and peripheral mononuclear cells are taken and processed according to the guidelines published in 2015 [85]. Samples are stored in our GERO biobank for long-term storage at − 80 °C or in liquid nitrogen (see details in annex). Stool samples are being collected using standardized kits and DNA extracted using the protocol Q suggested by the international human microbiome standards (IHMS SOP 06 V1). Data are recorded in an ad-hoc platform developed by bioinformatics and bioengineers personal of GERO (see Additional file 1). A schematic representation of instruments and assessments is presented in Table 1.

**Table 1:** Schedule of enrolment, assessments and close-out

		Enrolment	Assessment		Close-out
**TIMEPOINT**		***-t***_***1***_	***t***_***1***_	***t***_***2***_	***t***_***3***_
**ENROLMENT:**					
***Eligibility screen*** ***Informed consent***		X			
		X			
**ASSESSMENTS:** ***Functionality***	Technological - Activities of Daily Living Questionnaire (T-ADLQ) [30].	X		X	X
	Everyday Cognition Scale (ECog) [31].	X		X	X
	Pfeffer Functional Activities Questionnaire (PQAF) [29].	X		X	X
***Socio-demographic*** ^***a***^ ***[67]***	Marital status.		X	X	X
	Education.		X		
	Occupational background.		X		
	Ethnicity.		X		
	Individual and household income.		X	X	X
	Assets inventory.		X	X	X
	Health insurance.		X	X	X
	Household conformation.		X	X	X
	Social network information.		X	X	X
***Health, risk factors, anthropometric and laboratory assessment*** ^***b***^	Health related quality of life (EQ-5D) [32].		X	X	X
	Tabacum and alcohol consumption (Alcohol Use Disorders Identification Test, AUDIT) [33].		X	X	X
	Audition and vision section of the Chilean National Health Survey [34].		X	X	X
	Physical activity, sedentarism and diet [34].		X	X	X
	Oral health thought the Oral Health Impact Profile (OHIP).		X	X	X
	Frailty: Fried Frailty Phenotype and the Frail Questionnaires [35, 36].		X	X	X
	Anthropometric measurements: weight, body mass index (BMI), systolic and diastolic blood pressure (seat and standing).		X	X	X
	Framingham Cardiovascular Risk Scale.		X	X	X
	Laboratory evaluation: hemogram, glycaemia, lipid profile, level of vitamin B12 and folic acid, thyroid hormone (TSH and free T4) and hepatic profile.		X	X	X
	Health inventory on 18 health conditions (including cardiovascular events).		X	X	X
***Psychological assessment*** ^***c***^	Engagement in stimulating activities.		X	X	X
	Ageing related losses.		X	X	X
	Personality traits [37, 38].		X	X	X
	Psychological well-being [39].		X	X	X
	Geriatric Depression Scale - Brink and Yesavage [40].		X	X	X
	Depression, Anxiety and Stress Scale (DASS-21) [41].		X	X	X
	Coping processes [42].		X	X	X
	Social integration.		X	X	X
	Cognitive reserve scale [43].		X	X	X
***Stage and clinical symptoms*** ^***d***^	Clinical Dementia Rating for Frontotemporal Lobar Degeneration (CDR-FTLD)-eight domains [44].	X		X	X
	Alzheimer Disease- 8 (AD8) [45, 46].	X		X	X
***Neuropsychological evaluation***	Global Cognitive Function:				
	- Minimental-State Examination (MMSE) [47].	X		X	X
	- Montreal Cognitive Examination (MoCA) [48].	X		X	X
	- Addenbrooke’s Cognitive Examination (ACE III) [49].		X	X	X
	Memory: - Short Term Memory Binding Test [50, 51]. - Free and Cued Selective Reminding Test (FCRST) [52–54] - Supermarket task [55].		X	X	X
	Executive functions: - Ineco Frontal Screening [56]. - Verbal fluency test [57]. - Color Trail Test Part B [58, 59].		X	X	X
	Language: Sydney Language Battery (Sydbat) [60].		X	X	X
	Visuo-constructive abilities: Rey Complex Fig [61, 62]..		X	X	X
	Social Cognition: MiniSea [63].		X	X	X
***Motor assessment*** ^***e***^	Soft neurological signs: -Heidelberg Neurological Soft Signs [64]. -Edinburgh Motor Assessment (EMAS) [65].		X	X	X
	Balance: simple-task, dual-task (including cognitive task), and sensorimotor task.		X	X	X
	Walking assessment: carrying a cup with water, and counting backwards from 100.		X	X	X
	Other scales: - Tinetti test [66]. - Activities-Specific Balance Confidence Scale (ABC) [67]. - Timed up and go [68].		X	X	X
***Neuroimaging*** ^***f***^	Three whole-brain sequences: - High-resolution T1-weighted magnetic resonance image (MRI). - Resting-state functional magnetic resonance images (RS-fMRI) - Diffusion tensor-based images (DTI). - Axial T2 and Flair sequences to detect infarcts and white-matter alterations.		X		X
***Gut microbiome***	16S analysis from stool samples [69].		X	X	X
***Biomarkers***	Six inflammatory biomarkers, IL-2, IL-6, IL-10, TNFα, SAP and CRP [70–74].		X		X
***Genetic study***	Family pedigree through a questionnaire in accordance to Goldman criteria [75].		X		
	Candidate genes associated with neurodegenerative diseases (ApoE, TREM2 and MAPT) using real time PCR analysis.		X		
	Genome-Wide Association Study (GWAS) [76].		X		

^a^ This module used standard items taken from previous studies [34]

^b^ Chile has its own prices to valuate health states using EQ5D [32, 77]. Items for tabacum consumption, physical activity, sedentarism, diet evaluation were taken from the National Health Survey 2009–2010, many of them in accordance to PAHO monitoring instruments [34]. AUDIT instrument has been validated in Chile [33]. Health inventory includes items for diagnosis, past and current treatment [78]. Operational measure of frailty includes a brief 5 items scale: unintentional weight loss, weakness, exhaustion, slow gait, and low physical activity [79, 80]. Framingham scale (validated in Chile) includes diabetes, hypertension, dyslipidemia, tabacum consumption, male gender and age as risk factor of cardiovascular disease [81]

^c^ Instruments previously validated for the Chilean population. Instruments developed by GERO (engagement in stimulating activities, aging related losses and social integration) and validated in a pilot study with a sample of 250 elderlies

^d^ AD8 has been validated in Chile [82]

^e^ Balance is evaluated using a Bertec FP4060–05-PT force platform (Bertec Corporation, Columbus, Ohio, USA). Electro-cardio-physiological and electrodermal activity is collected through a BIOPAC MP150 device (BIOPAC Systems Inc., Goleta, CA, USA). A custom-made MATLAB script is used to present the stimuli and send triggers to the AcqKnowledge software (BIOPAC) in sync with the onset of the stimuli

^f^ For a more detailed information of the neuroimaging protocol see Additional file 1

### Data analysis plan

The GERO cohort offers a unique opportunity for multiple analyses to identify, correlate and analyze multidimensional factors related to FD and progression to dementia in elderlies with SCC.

In broad terms, a descriptive of baseline measurements (either outcomes or potential predictive factors) will be performed. The procedure will be repeated at each measurement time, every 18 months. Random effect models will be used for describing trajectories of participant subgroups and the whole cohort according to main variables, using Markov-Chain Montecarlo procedures [86, 87].

The association between variables and outcomes will be explored broadly using different machine learning methods, such as elastic net procedure, random forest procedure, based-tree methods, and support vector machines [88]. These procedures are suitable for leading with multi-collinearity and also high dimensional data (e.g. the number of predictive variables is larger than the number of participants in the cohort). Interpretation of causality will be conducted using standard random effect models and eventually structural equation modelling [89].

Missing data and loss of follow up of participants are common in observational studies, mainly in cohorts. Firstly, cases with missing data in any outcome will be explored and compared with cases without missing data describing any pattern. Secondly, two strategies will be followed to estimate results: i) to analyze only cases with complete information (i.e. assuming that missing data is completely at random); and ii) imputing data according to multivariate imputation by chained equation techniques [90, 91]. The analysis will be performed using the statistical software R.

### Coordination with local health services

The GERO cohort has been carefully designed to avoid undermining the usual care of participants in their common health services facilities. Even more, a linkage between the health assessments provided by the cohort and the usual health care has been promoted.

In cases when the cohort’s assessment detects a new health condition (diabetes, depression, hypertension, etc.) the participants are derived to the primary healthcare center of their territory. In the case of detection of a significant neurological disorder (Dementia syndrome, Parkinson, etc.) the participants are directly derived to specialized care according to their Health District, communicating the decision to the primary health care.

Primary care health centers, specialized care polyclinics and the direction of the Health District involved have been informed about the study and jointly the protocol of derivation and communication were established.

### Regulation of access to data/biospecimens

The access to data and biospecimens is regulated by the GERO directorate in accordance with the local Institutional Review Board authorization. A bilateral agreement must be signed before sharing of data. Access to the server will not be granted.

### Ethics

The project was approved September 2016 by the Ethic Committee of the Servicio de Salud Metropolitano Oriente, Santiago (Chile). A written informed consent to participate in the study is obtained for all participants of the GERO cohort.

### Outreach/dissemination and clinical impact of the GERO cohort

Our group, in collaboration with the Ministry of Health, the Hospital del Salvador and other faculties of the University of Chile, created in 2018 the CMYN, a clinical facility that houses one of the three Memory Units of the Chilean’s Dementia Plan, and it is conformed by a multidisciplinary team (neurologist, psychiatrist, nurse, neuropsychologist, clinical psychologist, occupational therapist, speech therapist and social worker). Nowadays, GERO and CMYN train professionals in primary care centers and neurology, psychiatric and geriatric residents in brain ageing and dementia. Additionally, we perform outreach activities on geroscience, brain ageing, and dementia to the broad community, mainly in the three districts of the GERO cohort and in Hospital Salvador, and to the scientific and health community. We designed a brochure to inform about the GERO cohort and performed broader dissemination through media (print, television, radio). Finally, our group lead a policy paper on dementia to inform public policy [92].

## Discussion

The current paper presents the study protocol of a Chilean cohort in brain aging and dementia: the GERO cohort study. This project mainly focuses in identifying risk factors associated with functional decline and progression to clinical dementia in the elderly with SCC by determine factors related to biomedical, clinical and psychosocial variables.

To date, important contributions have been realized in the Latin-America region allowing to know the prevalence and incidence of dementia [7, 10, 93], the subjective memory complaints in people with and without dementia [94], the neuropsychiatric symptoms as a risk of dementia [95], the unawareness of memory impairment in dementia [96], and biomarkers profiles in AD and MCI [97] in non-Caucasian population. However, study on the risk to conversion to dementia in elderly subjective cognitive complaint and MCI had been performed mainly in North America and Europe [98–107]. Epidemiologic studies of pathologic brain aging, SCC and MCI from Latin America and in particular Chile are still scarce in comparison with those from northern countries [9] and none of them have addressed risk factors related to FD, and its relationship with the progression to dementia. The transference of data collected from longitudinal and transversal European and North American studies to Latin-America population is limited due to the important differences in genetic, medical and social factors associated with FD and the risk of Dementia. In this context, the study of risk factors associated with dementia in non-Caucasian population has emerged as priority area in research [108]. GERO cohort address an under represented population in the literature [9].

From a public health perspective, identification of SCC and MCI subjects with higher risk of pathologic trajectories would be valuable as a diagnostic tool to focus prevention in subjects at higher risk of FD and dementia. Additionally, between SCC and MCI, SCC could be a better valuable target for public policy interventions, since it is built on the subjective self or informant-reported perceptions, which are closer to the awareness that could target health care consultation. Moreover, the diagnosis of MCI required the demonstration of an objective decline in cognitive performance limiting the diagnosis of MCI in primary care centers. Also, the concept of MCI has been criticized due to overlap of MCI and AD dementia suggesting ambiguity in the MCI concept and the criteria of MCI are continuing evolving [22].

The collective approach will allow us to i) improve diagnosis for neurodegenerative disease, ii) evaluate age-related risk factors and genetic variations linked to neurodegeneration, iii) understand how molecular mechanisms involved in aging lead to neurodegeneration and iv) explore novel biomarkers to evaluate the onset and progression of neurodegeneration. GERO’s aim is to establish a center for studying brain aging in Chile, including basic, translational and clinical research.

The availability of a GERO biobank will allow fostering translational studies by collecting peripheral samples for research use to improve our understanding of health and disease in the Chilean population. Additionally, biological analysis and associated clinical data are necessary to contribute in early diagnosis, prognostic and treatment for the aging population [14]. The development of and ad-hoc platform constitute an important step to the development of a brain aging registry in Chile who could contribute to advance in research in brain aging by collecting either epidemiologic data and data from other sources, such us from clinical practice with patients with brain disorders [109]. GERO translational approach combines basic and clinical scientists who are targeted to fills the void in aging research that exists in our country, specifically toward the interface between aging and neurodegenerative diseases.

Furthermore, GERO platform will provide strategies, methods and tools to conduct longitudinal studies on a community base in populations with diverse epidemiological settings.

### Main strengths and weakness of the GERO cohort

The main strengths of the GERO cohort is recruitment of participants at their home, allowing recruiting either people attending clinical center and people not attending. People not attending clinical center probably represent a higher risk group under-represented in previous studies [110]. Moreover, feasibility of memory clinical-based study in Latin America is limited, due to the important barriers to the diagnosis of dementia in Latino-America and generally consultation for memory problems occur in late stage of dementia disorder [9]; second, GERO cohort implemented a multidimensional-based evaluation, categorized in five main levels: i) biomarkers, ii) neuroimaging, iii) clinical phenotype (cognition, neuropsychiatric, motor and functionality), iv) metabolic, systemic diseases, and habits and v) psychosocial. This multidimensional approach is in line with evidence explaining FD of older adults with cognitive impairment by multiple factors [111]. Dementia also is umbrella term that include several diseases with important variability of genetic, neural, and behavioral manifestations [112], therefore a multilevel approach including molecular biomarkers, neuroimaging, genetic and clinical phenotypic allow a better characterization [25]. Third, we will explore predictive algorithms that will eventually predict rates of FD and conversion to dementia [113–115]. The development of bioinformatics and modelling algorithms during data analysis will allow the integration of complex data from multiple sources to build a comprehensive interaction model in our local aging population, which expect to uncover complex determinants of aging and brain diseases. Finally, establishing comprehensive databases for studies on aging can create the opportunity to formulate and validate tools for early detection of people who are at increased risk of late-life cognitive impairment, to identify important targets (risk factors) for preventive interventions, and to test such interventions in randomized control trials.

### Potential limitations

One of the main limitations of our study is a relatively small sample size. Due to our research strategy to prioritize a multidimensional and extensive evaluation, time and budget constraint, we limited the cohort size. Nevertheless, we selected a continuous outcome and will study the rate of change rather than a categorical outcome that allow to overcome possible limitations due to the sample size [22, 116]. Also due to budget constraint, we do not include determination of specific biomarkers for Alzheimer’s disease in spinal fluid and with PET (Pet amyloid and tau) [117], nevertheless we store blood sample that will allow to study blood-based biomarkers when available. Finally, as explained above, we have implemented a strategy to avoid limitation associated with attrition and missing data.

### Final message

Our work will allow us to determine multidimensional risks factors associated with the prognosis of elderly with cognitive complaint on functional decline in Chilean population. The GERO cohort will help to design public health policies tailored to prevent aging disease, and contribute to a better understanding of cognitive impairment and dementia in Latin America and the world. GERO’s aim is to establish a center for studying Brain Ageing in Chile including basic and clinical research.

## Supplementary Information

**Additional file 1.**

## Data Availability

Not applicable.

## Abbreviations

AD: Alzheimer’s disease
ADL: Activities of daily life
ANID: National Research and Development Agency (ANID, for its acronym in Spanish)
CDR-FTLD: Clinical dementia scale - frontotemporal lobar degeneration
CMYN: Memory and Neuropsychiatry Clinic (CMYN, for its acronym in Spanish)
DSM-V: Diagnostic and statistical manual for mental disorders fifth edition
FONDAP: Fund for research centers in priority areas program
GERO: Geroscience Center for Brain Health and Metabolism FD Functional decline
LA: Latin America
MCI: Mild cognitive impairment
MMSE: Mini-mental State Examination
MRI: Magnetic resonance neuroimaging
OR: Odd’s ratio
SCC: Subjective cognitive complaint
