# Supplementary material for: GERO Cohort Protocol, Chile, 2017–2022: Community-based Cohort of Functional Decline in Subjective Cognitive Complaint elderly
Source: BMC Geriatr. 2020 Nov 25;20:505. doi: 10.1186/s12877-020-01866-4 (PMC7690082; doi:10.1186/s12877-020-01866-4)
Supplement: Supplementary file 1 — Additional file 1. [file 12877_2020_1866_MOESM1_ESM.docx]

**GERO cohort Protocol, Chile, 2017-2022: Community-based cohort of Functional decline in Subjective Cognitive Complaint elderly**

**Additional File 1**

**Neuroimaging protocol**

- **T1 Magnetization prepared rapid gradient-echo (MP-RAGE):** whole-brain, acquisition parallel to the plane connecting the anterior and posterior commissures, repetition time (TR) = 1710 ms; echo time (TE) = 2000 ms; flip angle = 8º; 208 slices, matrix dimension = 224 x 224; voxel size = 1 x 1 x 1 mm^3^; sequence duration = 6 minutes.
- **Functional images (resting-state):** whole-brain, acquisition parallel to the anterior-posterior commissures, covering the whole brain, were sequentially and ascendingly acquired with the following parameters: TR = 2500 ms; TE = 30 ms; flip angle = 90º; 43 slices, matrix dimension = 74 x 74; voxel size in plane = 3 mm x 3 mm; slice thickness = 3 mm; sequence duration = 10 minutes; number of volumes = 300. Participants are request to keep their eyes closed, avoid moving, and to think in nothing in particular.
- **DTI:** whole-brain diffusion images with a twice-refocused, single-shot, echo-planar imaging pulse sequence, in the same plane as the MP-RAGE images. We used the following parameters: TR = 10000 ms; TE = 90 ms; flip angle = 90º; 76 slices, matrix dimension = 112 x 112; voxel size = 2 x 2 x 2 mm^3^; sequence duration = 10 minutes. The tensor was computed using 32 non-collinear diffusion directions (b = 1000 s/mm2) that were maximally spread by considering the minimal energy arrangement of point charges on a sphere, and one scan without diffusion weighting (b = 0 s/mm2, b0).
- **FLAIR**. Total scan duration: 3:14 min. Rel. signal level (%): 100. Act. TR/TI (ms): 8000. Act. TE (ms): 90.  ACQ matrix M x P: 175x320.  ACQ voxel MPS (mm): 0.7x0.7x4.  REC voxel MPS (mm): 0.7x0.7x4. Scan percentage (%): 70.  Min. slice gap (mm): 0. Optimal slices: 27. Max. slices: 58. WFS (pix) / BW (Hz): 289 Hz/Px. SAR/whole body: normal mode. Coil selection: Head/Neck Coil. FOV (mm): 220. Voxel size: 0.7x0.7x4. Slice thickness (mm): 4. Slices: 27. Slice gap (mm): 1. Slice orientation: transverse. Acquisition mode: IR. Fast Imaging mode: Turbo. Shot mode: 11. TSE factor: 16. Angle (deg): 150
- T2 AXIAL (name of the sequences: t2_tse_tra) will be acquired with the following parameters: Matrix size 435 x 512; TR= 5170ms; TE= 95ms; flip angle=150º, acquisition time=2.05 minutes.

**Biomarkers description**

Gero biological samples are taken and stored according to the guidelines published in 2015[1]. Samples were taken between 9 A.M. and 11 A.M. and peripheral blood was processed within 2h. Whole blood, buffy coat, plasma, serum and peripheral mononuclear cells were stored in 8 or 10 aliquots per patient. Subsequently, blood samples will be kept in a biobank for long-term storage at -80 °C or in liquid nitrogen at the Faculty of Medicine of the University of Chile. The samples are coded with a unique code that allows their identification and stored in the Gero record platform. After whole blood processing, we will analyze. To assess inflammatory biomarkers we will analyze six different inflammatory biomarkers in the Gero cohort serum, IL-2, IL-6, IL-10, TNFα, SAP and CRP. The analysis will be performed using Luminex platform at the University of Chile. These inflammatory biomarkers included cytokines, pentraxins and acute phase proteins, which have been extensively studied and differences in their expression levels have been observed between Alzheimer's patients and healthy controls [2-6].

**Record Platform**

Among available platforms for registry and analysis (see for example[7]), we have taken the approach of developing an ad-hoc platform taking ideas from other platforms, in particular KNIME [8]. Our platform covers data acquisition and also basic statistical data analysis. We designed a three-layered model composed by a database layer, a service layer, and a presentation layer. The platform was hosted on a WEB APACHE HP server with PHP support. Data security and integrity is guaranteed by a two mirrored-disks with Ubuntu 14.06 and data anonymity barriers. The database was developed using POSTGRESQL[9], whose NoSQL structure makes possible the integration of disparate data. The presentation layer, to apply surveys, save and explore the data, was implemented with JavaScript and it is accessed through a user-friendly WEB browser

References

1. O'Bryant SE, Gupta V, Henriksen K, Edwards M, Jeromin A, Lista S, Bazenet C, Soares H, Lovestone S, Hampel H *et al*: **Guidelines for the standardization of preanalytic variables for blood-based biomarker studies in Alzheimer’s disease research**. *Alzheimers Dement* 2015, **11**:549 - 560.

2. Araujo DM, Lapchak PA: **Induction of immune system mediators in the hippocampal formation in Alzheimer's and Parkinson's diseases: selective effects on specific interleukins and interleukin receptors**. *Neuroscience* 1994, **61**(4):745-754.

3. D'Anna L, Abu-Rumeileh S, Fabris M, Pistis C, Baldi A, Sanvilli N, Curcio F, Gigli GL, D'Anna S, Valente M: **Serum Interleukin-10 Levels Correlate with Cerebrospinal Fluid Amyloid Beta Deposition in Alzheimer Disease Patients**. *Neurodegener Dis* 2017, **17**(4-5):227-234.

4. Kim YS, Lee KJ, Kim H: **Serum tumour necrosis factor-alpha and interleukin-6 levels in Alzheimer's disease and mild cognitive impairment**. *Psychogeriatrics* 2017, **17**(4):224-230.

5. Tennent GA, Lovat LB, Pepys MB: **Serum amyloid P component prevents proteolysis of the amyloid fibrils of Alzheimer disease and systemic amyloidosis**. *Proc Natl Acad Sci U S A* 1995, **92**(10):4299-4303.

6. Gong C, Wei D, Wang Y, Ma J, Yuan C, Zhang W, Yu G, Zhao Y: **A Meta-Analysis of C-Reactive Protein in Patients With Alzheimer's Disease**. *Am J Alzheimers Dis Other Demen* 2016, **31**(3):194-200.

7. Canuel V, Rance B, Avillach P, Degoulet P, Burgun A: **Translational research platforms integrating clinical and omics data: a review of publicly available solutions**. *Briefings in bioinformatics* 2015, **16**(2):280-290.

8. Tiwari A, Sekhar AKT: **Workflow based framework for life science informatics**. *Computational Biology and Chemistry* 2007, **31**:305 - 319.

9. Ngari MM, Waithira N, Chilengi R, Njuguna P, Lang T, Fegan G: **Experience of using an open source clinical trials data management software system in Kenya**. *BMC Res Notes* 2014, **7**:845.
